# Epigenetic Drivers of Pulmonary Hypertension: Environment Meets Genome

**DOI:** 10.7759/cureus.104241

**Published:** 2026-02-25

**Authors:** William N Whitley, Richard M Millis

**Affiliations:** 1 Department of Basic Sciences, University of Health Sciences Antigua, St. John's, ATG; 2 Department of Physiology, American University of Antigua, St. John's, ATG

**Keywords:** environment-gene interaction, epigenetics, molecular biomarkers, pulmonary hypertension, risk factors

## Abstract

Pulmonary hypertension (PH) is a progressive disease in which the pulmonary arteries thicken and narrow, raising pulmonary vascular resistance (PVR) and eventually straining the right ventricle. Known gene mutations explain only a minority of cases and often do not account for why the disease starts, worsens, or varies so widely between patients. Growing evidence suggests that epigenetic changes, chemical marks on DNA and its packaging that alter how genes are used without changing the DNA sequence, help explain this gap. These changes, including DNA methylation, histone modification, and non-coding RNAs, can be triggered by common exposures and disease states, and they can produce lasting shifts in vascular, immune, and metabolic pathways.

This narrative review synthesizes current data showing how intrinsic stresses (mitochondrial dysfunction, oxidative stress, and cancer-like metabolic reprogramming) interact with extrinsic and often modifiable factors. Obesity, cigarette smoke, asbestos exposure, chronic hypoxia, and systemic inflammation drive PH through epigenetic reprogramming. We highlight major molecular hubs implicated across studies, including bone morphogenetic factor receptor 2 (BMPR2), NOTCH3, endothelin-1 (ET-1), transforming growth factor‑β (TGF-β), interleukin‑6 (IL-6), and CCL5, and we summarize emerging therapeutic approaches aimed at epigenetic regulators and microRNA networks.

This narrative review was not conducted under Preferred Reporting Items for Systematic Reviews and Meta-Analyses (PRISMA) guidelines and does not constitute a formal systematic review. The information in this review provides a practical framework for clinicians and researchers to improve risk assessments, to employ biomarkers, and to develop therapies that go beyond vasodilation to address upstream drivers of pulmonary arterial remodeling. This framework may also serve as a model for other difficult-to-treat diseases in which incomplete genetic explanations and limited attention to environmental exposures have slowed progress in prevention, early detection, and personalized treatment.

## Introduction and background

Literature search strategy

This narrative review was performed using structured searches of PubMed/Medical Literature Analysis and Retrieval System Online (MEDLINE), Scopus, and Google Scholar through December 2025, expanded to January 2026 for clarifications based on adviser and reviewer critiques. The search terms included combinations of “pulmonary hypertension,” “pulmonary arterial hypertension,” “epigenetics,” “DNA methylation,” “histone modification,” “microRNA,” “mitochondrial dysfunction,” “oxidative stress,” and “environmental exposure.” We prioritized peer-reviewed original studies, mechanistic investigations, translational research, and high-quality review articles relevant to pulmonary vascular remodeling. As a narrative review, this study was not conducted under Preferred Reporting Items for Systematic Reviews and Meta-Analyses (PRISMA) guidelines and does not constitute a formal systematic review or meta-analysis.

Epidemiology of pulmonary hypertension (PH)

PH is a heterogeneous cardiopulmonary disorder defined hemodynamically by an abnormal elevation in mean pulmonary arterial pressure (mPAP) measured by right heart catheterization; contemporary consensus defines PH as mPAP >20 mmHg at rest and, for pre-capillary PH, also requires pulmonary vascular resistance (PVR) ≥3 Wood units with a pulmonary arterial wedge pressure ≤15 mmHg [1, 2]. Epidemiologically, PH is divided into a spectrum of conditions with varying prevalence (Groups 1-5). Group 1: Pulmonary arterial hypertension (PAH), where there is intrinsic remodeling of pulmonary arteries; Group 2: PH due to left heart disease; Group 3: PH due to lung disease and/or hypoxia; Group 4: PH due to pulmonary artery obstruction; and Group 5: PH involving unclear or multifactorial mechanisms. When all groups are considered, including PH due to left heart disease and chronic lung disease, the condition affects approximately 1% to 2% of the general population, with prevalence rising in individuals over 65 years of age. In contrast, PAH, the pre-capillary form and the subtype, has a reported incidence of approximately one to seven cases per million annually and a prevalence ranging from 10 to 50 cases per million. PH is predominant in females, with female-to-male ratios typically between 2:1 and 4:1. Connective tissue diseases, particularly systemic sclerosis, represent a major associated condition; PH develops in approximately 8% to 12% of systemic sclerosis patients and accounts for a significant proportion of associated PH cases, indicating a correlation between immune dysregulation and pulmonary vascular pathology. Trends over recent decades suggest increasing absolute case numbers, likely attributable to improved recognition and demographic aging, while survival has improved with advances in targeted therapy [3].

Regardless of etiology, sustained elevation in right-ventricular (RV) afterload promotes RV hypertrophy and maladaptive remodeling that can progress to RV failure, a major determinant of outcomes [1]. Early manifestations (e.g., exertional dyspnea, fatigue, chest discomfort) are nonspecific, contributing to delayed recognition; in the Registry to Evaluate Early and Long-Term PAH Disease Management (REVEAL) registry, approximately one in five patients with PAH reported symptoms for >2 years before diagnosis [4]. Although pathogenic variants, such as BMPR2, represent the most common genetic cause of heritable PH, known mutations explain only a minority of sporadic cases and exhibit incomplete penetrance, implying additional modifying influences [5]. Epigenetic mechanisms (DNA methylation, histone modifications, and non-coding RNAs) provide a biologically plausible bridge by which environmental exposures, inflammation, hypoxia, and metabolic stress may contribute to durable changes in gene expression that shape pulmonary vascular remodeling and clinical heterogeneity in PH.

Pathophysiology of PH

PH remains a progressive condition with significant morbidity and mortality, with contemporary registry data demonstrating an approximate 21% mortality within three years of diagnosis [6]. A problem with diagnosing and treating PH is that it is commonly asymptomatic in the early stages, and when symptoms begin to appear, they are consistent with multiple other respiratory and cardiac conditions, thereby resulting in delayed diagnosis and treatment [7]. Delayed diagnosis of pulmonary hypertension contributes significantly to morbidity and mortality. A combination of non-invasive tests can aid earlier detection: chest X-ray (CXR) may reveal enlarged pulmonary arteries or right heart enlargement, arterial blood gas (ABG) analysis can show hypoxemia or hypocapnia, and electrocardiography (ECG) may demonstrate signs of right ventricular hypertrophy or strain. Transthoracic echocardiography is recommended as the primary screening modality for estimating pulmonary artery pressure and assessing right heart structure and function. In addition, elevated brain natriuretic peptide (BNP) or N-terminal pro-BNP (NT-proBNP) concentrations, reflecting RV strain, can further support clinical suspicion of PH and help prioritize patients for definitive investigations such as right heart catheterization [8]. The hallmark of PH is vascular remodeling, which increases PVR and leads to eventual right ventricular heart failure. Epigenetic data are beginning to create some understanding of the potential pathobiological mechanisms of idiopathic PH (IPH). This review, therefore, aims to highlight the fundamentals of epigenetic linkages to PH and iPH, which may help in reducing the delays in diagnosis and treatment of PH. 

Epigenetics

Epigenetics refers to chemical modifications of DNA or its associated histone proteins that regulate which genes are expressed and which are silenced in a cell. The regulating molecules are known as "marks" of previous or ongoing epigenetic regulatory activity. The three best-studied epigenetic marks are: (1) DNA methylation: a methyl cap on cytosine bases that usually silences genes; (2) Histone modification: acetyl or other groups on histone tails that loosen (activate) or tighten (silence) DNA; and (3) Non‑coding RNAs: small RNAs, such as microRNAs, that bind messenger RNA and block protein translation [9]. Because these marks are reversible and respond to diet, oxygen tension, toxins, and metabolism, they translate external stimuli into durable shifts in gene expression.

The two primary epigenetic mechanisms identified by the aforementioned "epigenetic marks" influence several external factors affecting gene expression without modifying a person’s genome [10]. (1) DNA methylation is one of two primary mechanisms for suppressing gene expression using DNA methyltransferase enzymes (DNMTs) to transfer a methyl group from S-adenosyl methionine (SAM). SAM is a metabolite derived from the nutritionally essential amino acid methionine, attached to the fifth carbon on the cytosine base of DNA. DNMTs increase the binding strength of DNA to its histones, thereby decreasing the gene’s potential for expression as proteins. The majority of methylations occur at the promoter regions of DNA containing the base cytosine, followed by the base guanine on single strands of DNA known as CpG islands. CpG islands are, therefore, highly expressed in silenced genes that are extensively methylated [11]. (2) Histone modification includes histone acetylation, involving the use of histone acetyltransferases (HATs) to transfer acetyl groups from acetyl-coenzyme A, a metabolite of the nutritionally essential B vitamin known as pantothenic acid. HATs transfer acetyl groups to the amino terminal of the nutritionally essential amino acid lysine, comprising the histone proteins, which affix DNA molecules by wrapping them around chromosomes. Gene expression produces gene products in the form of structural and functional proteins such as enzymes and membrane receptors, which, in turn, maintain cellular integrity as well as cell, tissue, and organ functions. A person’s heritable genetic traits are reflected in these proteins. In contrast to HATs, histone deacetylases (HDACs) remove acetyl groups from histones, condensing the chromatin. This decreases the expression of genes seen as the gene products, which are the proteins making up a person’s heritable traits. Deacetylation by HDACs involves the removal of acetyl groups from histones, allowing the histones to revert to their natural coiled heterochromatin form, which reduces the transcription potential of the gene and reduces gene expression. Imbalances in either of these two processes can cause deficiency or excess production of the structural or functional proteins encoded by a particular gene. (3) Non-coding RNAs are generally not considered a primary epigenetic mechanism. Unlike stable covalent DNA methylation and histone acetylation or methylation, non-coding RNAs act as transient regulatory guides that influence gene expression indirectly by blocking gene product (protein) translation rather than serving as durable, self-propagating chromatin marks. 

Aging, oxidative stress, and vascular remodeling

Numerous studies have highlighted the role of epigenetics in modulating gene expression in disease conditions. These insights underscore the importance of understanding how both genetic predispositions and external, largely environmental, factors contribute to the pathogenesis of complex diseases such as PH via epigenetic mechanisms. In one experimental rat study, hypermethylation of mitochondrial genes for producing cytochrome oxidase subunit 2 (COX2), not to be confused with cyclooxygenase-2, involved in pro-inflammatory prostaglandin synthesis, an enzyme involved in oxidative stress by amplifying the production of reactive oxygen species (ROS), was shown to be associated with senescent human cardiac mesenchymal stem cells (HMSCs). Such HMSCs are involved in the evolution of hypertension, heart failure, and thrombotic events [12]. Mitochondria are crucial for producing antioxidants that mitigate cell damage due to the excess production of ROS, which plays a significant role in vascular health. Although cytochrome oxidase complexes I and III are recognized as the principal mitochondrial ROS generators [13], changes in complex IV subunits may impair terminal electron transfer, increasing upstream electron leak and amplifying mitochondrial ROS signaling. Mitochondrial oxidative stress is a central driver of pulmonary vascular remodeling [14, 15]. Transcriptomic and functional analyses have identified mitochondrial oxidative stress-related hub genes, including complex IV-related subunits such as COX6B1, found to be upregulated in idiopathic PH, with experimental suppression reducing mitochondrial ROS accumulation and pulmonary arterial smooth muscle cell proliferation [16]. These findings support a model in which cytochrome oxidase complex IV changes modulate mitochondrial redox balance and contribute, albeit indirectly, to oxidative stress-dependent mesenchymal stem cell senescence and pulmonary arterial remodeling.

One key antioxidant enzyme produced by mitochondria is superoxide dismutase (SOD), which converts the highly reactive superoxide into hydrogen peroxide (H₂O₂), a less harmful molecule. This conversion is important for two reasons: (i) H₂O₂ is less likely to cause endothelial damage, and (ii) it plays a role in immune defense mechanisms, such as the respiratory burst in neutrophils, where ROS like H₂O₂ help kill pathogens [17]. Impaired SOD production or function can lead to excessive oxidative stress, contributing to endothelial damage and vascular remodeling. In the context of PH, increased ROS levels and endothelial dysfunction appear to be key factors in disease progression. The dysregulation of mitochondrial function, particularly through COX2 hypermethylation as cells age, can exacerbate oxidative stress and promote the vascular remodeling characteristic of PH. Additionally, mitochondria produce other antioxidants, such as glutathione and nitric oxide (NO), which further protect against oxidative damage. An imbalance in the production of these antioxidants due to epigenetic changes in COX2 during aging may worsen oxidative stress, accelerating aging-related cardiovascular conditions, including PH [17]. Oxidative stressors, DNA methylation, and histone acetylation are not only influenced by internal cellular processes but also by various external lifestyle factors. Chronic exposure to environmental stressors, such as cigarette smoke, whether through active or passive smoking, has been shown to induce hypermethylation in genes related to chronic obstructive pulmonary disease (COPD) and atherosclerosis, both of which are risk factors for PH [18].

Cardiac remodeling 

**Figure 1:**
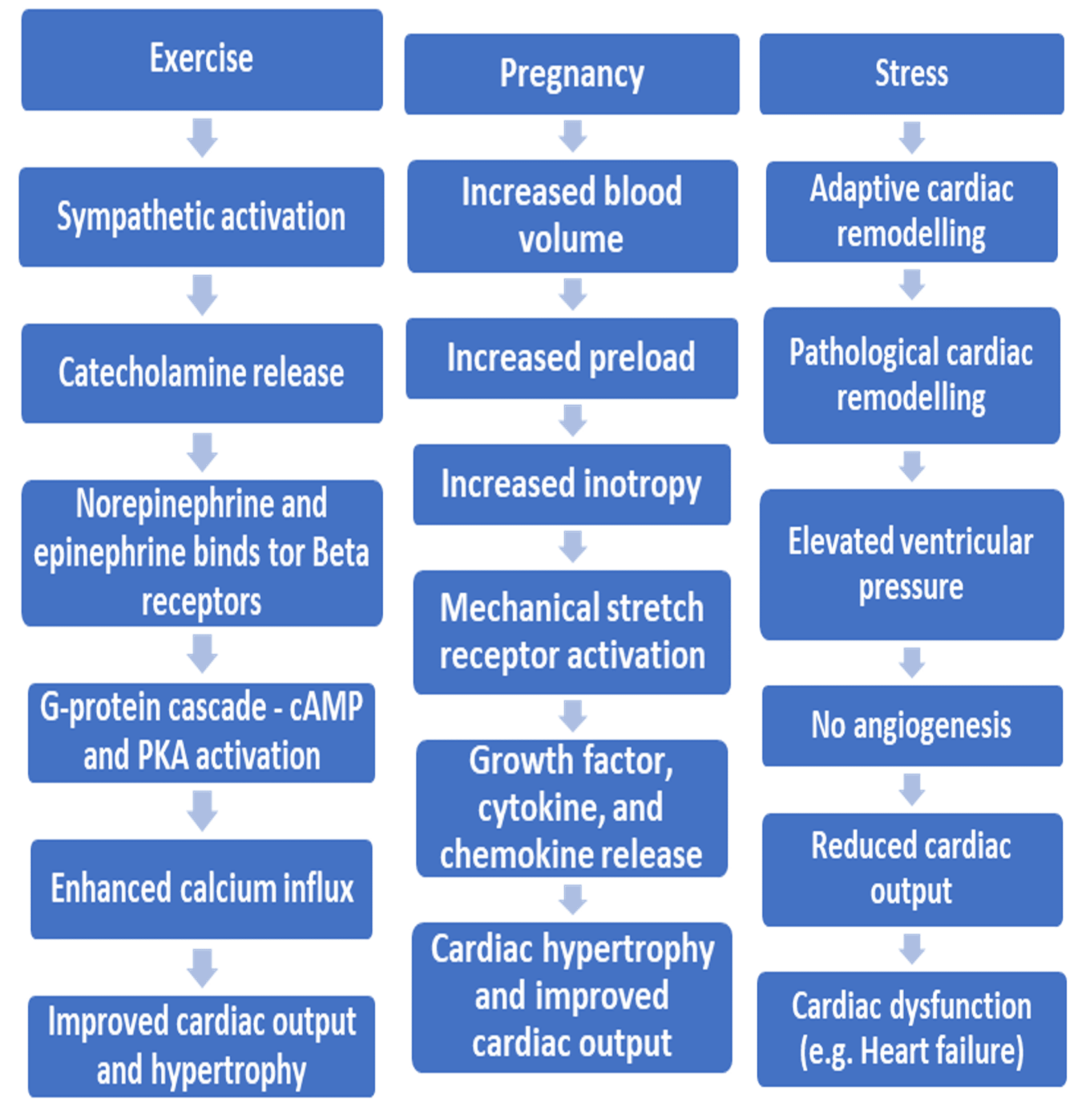
Flowchart depicting physiological and pathological cardiac remodeling

In response to physiological stressors, virtually all organs, including the heart, undergo remodeling to adapt to changing conditions. Figure 1 summarizes the mechanisms underlying cardiac remodeling adaptations, which can be physiological, such as during exercise or pregnancy, or pathological, as seen in conditions like PH.

Cardiac remodeling as a physiological adaptation

As depicted in Figure 1, physiological remodeling occurs to meet increased metabolic demands, such as the heightened need for oxygen and nutrients during exercise [19]. Exercise stimulates the sympathetic nervous system, increasing the release of catecholamines like norepinephrine. Norepinephrine binds to β-adrenergic receptors, activating the G-protein signalling cascade, which elevates intracellular cAMP levels and activates protein kinase A (PKA). PKA phosphorylates L-type calcium channels, enhancing calcium influx, which improves cardiac output and efficiency. Over time, this results in mild and often reversible cardiac hypertrophy, a beneficial adaptation to support increased inotropy during exercise. Pregnancy induces physiological cardiac remodeling due to a 40% to 50% increase in blood volume. This increased preload on the heart results in reversible cardiac hypertrophy to accommodate the higher stroke volume, in accordance with Starling’s law. The heart’s mechanical stretch receptors respond to this hemodynamic stress by releasing growth factors, cytokines, and chemokines. While this adaptation is typically reversible and normal during pregnancy, these signaling factors can also influence pathological conditions if the stress becomes excessive or persistent, potentially contributing to the development or progression of pulmonary hypertension [20, 21]. 

Pathological cardiac remodeling leading to heart failure 

Pathological cardiac hypertrophy occurs when adaptive cardiac remodeling, in response to stress, becomes maladaptive and is associated with cardiac dysfunction [22]. It is commonly linked to increased end-diastolic volume (preload) or increased systemic or pulmonary arterial pressure (afterload) and can serve as a precursor to heart failure. Pathological remodeling of the left ventricle leads to elevated pressures in the pulmonary circulation, contributing to PH. A key feature of physiological cardiac hypertrophy is an increase in angiogenesis and myocardial capillary density, which helps meet the increased metabolic demands of the cardiac muscle. However, in pathological remodeling, this compensatory increase in capillary density does not occur, impairing the heart's ability to meet its metabolic needs. Disruptions in angiogenesis may thus represent a critical point where physiological remodeling transitions into pathological remodeling. Signaling molecules like vascular endothelial growth factor (VEGF) play a key role in promoting angiogenesis, and disturbances in these molecules can contribute to pathological remodeling. A critical molecule in this process is p53, which inhibits angiogenesis. Studies have shown that the deletion of p53, a signaling molecule in endothelial cells with both antioxidant and pro-oxidant activities, the latter stimulating production of ROS, preserves capillary density, highlighting its role in angiogenesis. p53 also has the capacity to inhibit the transcriptional activity of hypoxia-inducible factor-1 (HIF-1), a key promoter of angiogenesis [23]. Another important mechanism in pathological remodeling is inflammation and oxidative stress. Proinflammatory cytokines, such as TNF-α, produced by macrophages, have been shown to be elevated in pathological cardiac remodeling and heart failure. TNF-α is a driver of cardiac fibrosis, impairing myocardial contraction. In that regard, TNF-α knockout mice exhibited reduced cardiac fibrosis following myocardial infarction [24]. This interpretation is supported by findings concerning the role of proinflammatory cytokines such as interleukin-6 (IL-6), IL-1β, IL-1RA, and TNF-α in activating nuclear factor-kappa B (NF-κB), all of which are hallmark features of pathological cardiac hypertrophy [25, 26]. These findings suggest that pathological remodeling is linked to the activation of proinflammatory signaling pathways mediated by ROS. These pathways, including tyrosine kinases (TK), protein kinase C (PKC), and mitogen-activated protein kinases (MAPK), have all been implicated in cardiac muscle hypertrophy and remodeling. 

Understanding pathological remodeling and treatment strategies 

Understanding pathological remodeling in PH can be aided by using the following cardiovascular system derivative of Ohm’s law to determine whether a condition is primarily influenced by cardiac or vascular factors: 

$\mathrm{mPAP} = \mathrm{CO} \times \mathrm{PVR}$

where mPAP represents mean pulmonary arterial pressure, CO is cardiac output, and PVR is pulmonary vascular resistance. Elevated MPAP and CO suggest that PAH may be influenced more by cardiac factors. Conversely, elevated MPAP and PVR indicate that the pulmonary vasculature plays a larger role in the condition. Identifying these influences is important for targeting appropriate treatments. 

The heart predominantly expresses β1-adrenergic receptors, which mediate chronotropic and inotropic responses. The pulmonary vasculature expresses α1, α2, β1, and β2 adrenergic receptors, with β2-mediated signalling contributing to physiological vasodilation. Although adrenergic pathways influence cardiac and vascular function in PH, α-adrenergic blockers are not used for treatment, and β-blockers are generally avoided in PAH as they may impair right ventricular output. Understanding this physiology is important as there is evidence to suggest that remodelling of the right ventricle from RV failure and PH is associated with a downregulation of β2 receptors [27].

To further distinguish between cardiac contributions, the following equation can be used to assess whether PH is influenced by stroke volume or heart rate: 

$\mathrm{mPAP} \approx \mathrm{dPAP} + \frac{1}{3} \left( \mathrm{sPAP} - \mathrm{dPAP} \right)$

where mPAP represents mean pulmonary arterial pressure, sPAP represents systolic pulmonary arterial pressure, and dPAP represents diastolic pulmonary arterial pressure. Increases in stroke volume will raise both sPAP and dPAP, but sPAP is more sensitive to changes in stroke volume, whereas dPAP is more influenced by changes in heart rate. Elevated mPAP with elevated sPAP suggests that treatments targeting stroke volume are needed, while elevated mPAP with elevated dPAP indicates that treatments should focus on heart rate. Understanding these relationships helps tailor treatments to specific factors contributing to PH, thereby improving therapeutic outcomes. 

The Warburg effect and epigenetic mechanisms in PH

**Figure 2:**
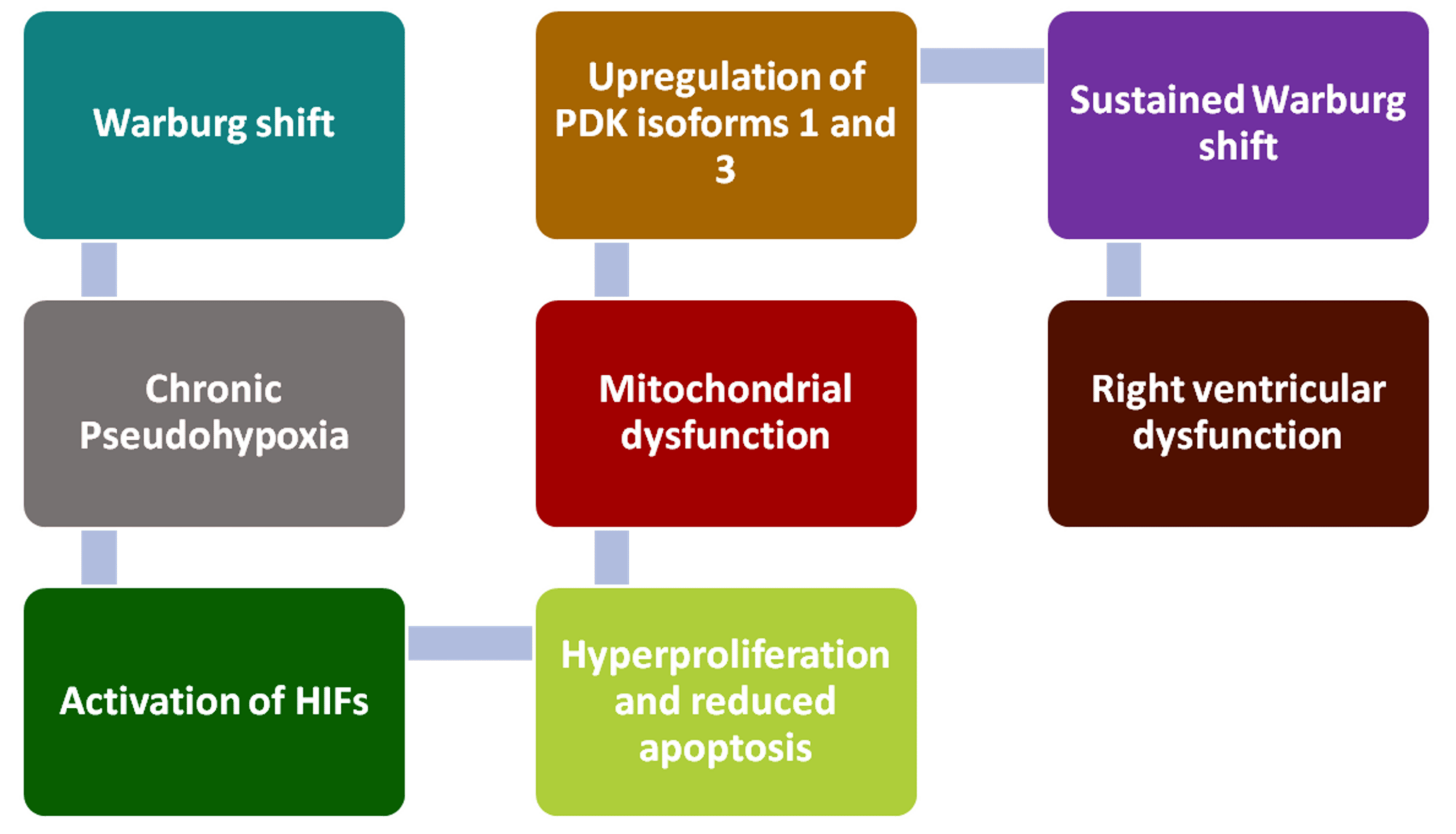
Impact of the Warburg effect on pulmonary hypertension

The Warburg effect involves a shift from mitochondrial aerobic metabolism of glucose to cytoplasmic anaerobic glycolysis even in the presence of sufficient oxygen. The Warburg effect is often observed in hyperproliferative cancer cells. This metabolic shift results in chronic “pseudohypoxia” and activates HIFs, peptides that promote cellular proliferation under conditions of reduced oxygen [7]. In PH, the Warburg effect is associated with hyperproliferation and apoptosis resistance of pulmonary arterial smooth muscle cells (PASMCs), pulmonary adventitial fibroblasts (PAfib), pulmonary artery endothelial cells, and RV cardiomyocytes. This metabolic shift, combined with reduced mitochondrial function and HIF signaling, contributes to endothelial dysfunction and cellular hyperproliferation. In the pulmonary vasculature and right ventricle. HIF-dependent signaling has been shown to favor resistance to apoptosis and maladaptive remodeling, which may contribute to hypocontractility of the right ventricle and progression to right ventricular failure. Additionally, the upregulation of pyruvate dehydrogenase kinase (PDK) isoforms 1 and 3 inhibits pyruvate dehydrogenase (PDH), impeding the conversion of pyruvate from glycolysis, sustaining the Warburg effect. Figure 2 shows how key steps of the Warburg effect impact the development of PH.

Epigenetic changes provide additional mechanisms for activating HIFs. PH also leads to the upregulation of DNMTs 1 and 2, resulting in hypermethylation of mitochondrial DNA (mtDNA), thereby disrupting the production of key enzymes such as SOD 2 (SOD2) [22]. This reduction in SOD2 activity impairs the detoxification of ROS, leading to increased superoxide levels and decreased hydrogen peroxide [12]. This disrupts mitochondrial redox signaling, promotes the activation of HIFs, and inhibits apoptosis, contributing to cell proliferation. These altered ROS levels result in vascular damage, which can be a significant factor in cardiac remodeling and in the development of PH. This damage to blood vessels, combined with the hyperproliferation of vascular and cardiac cells, leads to an accumulation of fibroblasts and excess production of collagen, leading to fibrosis. This fibrosis in the pulmonary vasculature and RV reduces vascular compliance and increases PVR, thereby exacerbating the symptoms of PH [28].

## Review

Risk factors that influence epigenetic changes in PH 

Obesity

Because adipose tissue is both an immunologic and endocrine tissue, adipocytes can upregulate inflammation driven by adipocytokines. A specific imbalance of adipocytokines, particularly reduced levels of adiponectin in obese individuals, contributes to endothelial dysfunction and oxidative stress involved with PH [29]. This decline in adiponectin leads to impaired production of the primary endothelial-derived vasodilator NO, thereby promoting pulmonary arterial vasoconstriction, increased vascular resistance, and elevated pulmonary artery pressure [30]. Additionally, the pro-inflammatory environment seen in obesity, characterized by elevated TNF-α and IL-6 levels, further exacerbates pulmonary vascular remodeling by fibrosis and smooth muscle proliferation. These combined effects not only accelerate the progression of PH but also increase the risk of RV heart failure. The epigenetic and metabolic changes in obesity appear to parallel the changes in molecular pathways known to be involved in PH. Targeting the effects of obesity, especially by preventing a decline in adiponectin, could provide a novel therapeutic approach to slowing the progression of PH. 

Smoking and PH

Even relatively brief periods (three to four months) of smoking are thought to contribute to PH [19]. One study shows that hypermethylation is associated with PH pathogenesis, particularly in regard to the aspect of pulmonary arterial remodeling [19, 28]. Results from this study showed that exposure to cigarette smoke increased DNMT1 protein levels involved with methylation. Another important protein that has been focused on was RASEF, part of a family of proteins known as the Rab family. Although research on this protein is limited, there have been links between exposure to cigarette smoke and reduced expression of RASEF due to hypermethylation. The reduced expression of RASER may promote pulmonary arterial smooth muscle cell proliferation and migration, contributing to vascular remodeling. Conversely, overexpression of RASEF has been shown to alleviate cigarette smoke-induced PAH, suggesting it may serve as a therapeutic target [7]. Additionally, cigarette smoke-induced hypermethylation of RASEF and increased expression of proteins like Rab5a, another member of the Rab family, have also been associated with smooth muscle cell proliferation and migration. Cigarette smoke-induced alterations to epigenetic processes may persist even after smoking cessation, suggesting a long-term impact on pulmonary vascular remodeling that continues to drive disease progression. While research on RASEF and other Rab proteins is still emerging, when overexpressed, its role in alleviating PH highlights the potential for targeting RASEF and Rab proteins therapeutically in the context of cigarette smoke-induced PH.

Asbestos and PH

Exposure to chemicals, such as asbestos, is another risk factor for PH. Because of the abundance of asbestos in old housing, it can be a factor used to identify a PH risk group. Despite the ban on asbestos use in the UK in 1999, it still exists in a significant proportion of the country’s infrastructure [31]. According to the UK Health Security Agency, the construction industry is particularly affected. Risk factors such as the production of materials like insulation (prior to 1999), construction work involving asbestos removal or maintenance, and the transport and disposal of asbestos-containing materials all may contribute to the PH health cost burden [32]. Beyond occupational exposure, many individuals continue to live in homes that contain asbestos, contributing to their risk of developing PH and other pulmonary conditions. Identifying this data and risk factors allows us to identify high-risk groups, particularly those working in construction or living in older housing. Asbestos is a naturally occurring fiber that, when inhaled, induces an inflammatory response in the lungs. These fibers can persist in lung tissue for extended periods, causing the initial acute inflammation to progress to chronic inflammation [33]. This chronic inflammation can extend into the pulmonary vasculature, leading to vasoconstriction and damage to the blood vessels, contributing to PH. Additionally, asbestos directly damages the endothelial lining of the pulmonary vasculature, resulting in further injury and inflammation. One study supports these findings and highlights the mechanisms underlying this process [34]. Specifically, it explains how asbestos exposure activates the NLRP3 inflammasome within macrophages. Upon inhalation, macrophages phagocytose asbestos fibers, and NLRP3 is activated, leading to the production of pro-inflammatory cytokines such as IL-1β and IL-18. These cytokines are released into the lung tissue, promoting sustained inflammation. Prolonged exposure to asbestos and continuous activation of the NLRP3 inflammasome may, therefore, exacerbate chronic inflammation and contribute to the development of PH. 

The chronic inflammation induced by asbestos in the lung often results in pulmonary fibrosis. The excessive collagen deposits reduce the elasticity and compliance of the lung, which reduces the efficiency of gas exchange. Reducing ventilation causes a lower ventilation-perfusion (V/Q) ratio and results in hypoxemia. In response to lower V/Q areas of the lung, pulmonary vasoconstriction allows blood to be diverted to areas of higher V/Q ratios. In most cases, this is a reversible response to hypoxic areas of the lung, which reduces hypoxia. However, during prolonged hypoxia of areas of the lung, cardiac and vascular remodeling can occur, inducing PH [35]. Mitochondria within the smooth muscle of the arteries contribute to the detection of hypoxia and the redistribution of blood to lung areas of higher V/Q ratios.

High Altitude Hypoxia and PH

Chronic exposure to high altitude appears to induce epigenetic changes that contribute to PH. Cells isolated from vessel walls of patients diagnosed with PH exhibit hyperproliferation, resistance to apoptosis, increased inflammation, and fibrosis [36]. These alterations are associated with changes in DNA methylation, histone modification, and altered levels of miRNA. Specifically, miRNAs play a role in gene regulation by binding to the 3’ untranslated regions (UTR) of mRNA, inhibiting translation, or recruiting RNA-induced silencing complexes for mRNA degradation. The study highlighted that miR-17, miR-21, and miR-190 are upregulated in response to hypoxia at high altitudes. miR-17 promotes the proliferation of PASMCs, contributing to vascular remodeling. miR-21 is involved in PASMC migration, vascular fibrosis, and inflammation. Which reduces vascular compliance and exacerbates chronic inflammation and PH. miR-190 also contributes to vascular remodeling and vasoconstriction involved in PH. Additionally, significant downregulation of miR-124 has been observed in calves and humans with severe PH; overexpression of miR-124 correlates with the downregulation of fibroblast proliferation and migration [36]. Furthermore, reduced histone H4 acetylation in PASMCs from high-altitude-exposed fetal lambs correlates with decreased cyclin-dependent kinase inhibitor p21 and increased cell proliferation, contributing to PH. These hypoxia-induced epigenetic changes seem to play a significant role in the progression and severity of PH. Addressing hypoxia or targeting these epigenetic modifications may, therefore, offer novel therapeutic strategies. 

Chronic Inflammation and PH

Chronic inflammation, in and of itself, may also play a key role in influencing epigenetic changes. The aging of cells, which is known to increase inflammation, has been associated with aberrant DNA methylation of COX genes within mtDNA, contributing to conditions such as hypertension, heart failure, and thrombotic events [12]. Similarly, chronic inflammation in lung tissue drives epigenetic modifications through the production of ROS. The oxidative stress generated by ROS, alongside inflammatory mediators like IL-6 and TNF-α, enhances the activity of DNMTs such as DNMT1 and DNMT3a, while reducing HDAC activity. This results in the hypermethylation of key regulatory genes and decreased expression of protective genes in the pulmonary vasculature. One study further supports that elevated levels of DNMT1 and DNMT3a in chronic inflammation silence protective genes and upregulate genes involved in pulmonary fibrosis [37]. These fibrotic changes can extend to the pulmonary vasculature, contributing to vascular remodeling. Additionally, chronic inflammation-induced mitochondrial dysfunction increases ROS production, continuing a cycle of oxidative stress that drives further epigenetic alterations. Such changes are closely linked to pulmonary vascular remodeling, exacerbating the progression of PH. By understanding the epigenetic impact of chronic inflammation, we may identify potential therapeutic targets to mitigate these changes and slow the progression of PH. Table 1 summarizes the mechanisms by which inflammation and many other factors may contribute to the evolution of PH by rewriting the epigenome.

**Table 1:** Extrinsic triggers that rewrite the epigenome

Factor	Mechanism of Action	Key Epigenetic Changes	Impact on Pulmonary Hypertension	Key Studies
Obesity	Adipose tissue dysfunction and altered production of adipokines	DNA methylation (hypermethylation of key genes)	Inflammation, endothelial dysfunction	[7, 19]
	Inflammation-elevated cytokines (TNF-α, IL-6)	Reduced RASEF expression, gene hypermethylation	Atherosclerosis, vascular remodeling, and increased pulmonary vascular resistance	
	Endothelial dysfunction	Increased DNMT1 protein levels, miRNA changes	Pulmonary vascular remodeling, increased pulmonary vascular resistance	
	Atherosclerosis and altered lipid metabolism	Pulmonary vascular resistance and remodelling	Elevated pulmonary arterial pressure	
Smoking	Exposure to cigarette smoke	Increased DNMT1 protein levels	Pulmonary artery smooth muscle proliferation, migration, and vascular remodeling	[19, 28]
	Inflammatory response in the lungs	Activation of NLRP3 inflammasome	Increased inflammation, fibrosis, and epigenetic changes	
	Progressive inflammation leading to vascular remodeling	Increased pulmonary arterial smooth muscle cell proliferation and migration	Elevated pulmonary arterial pressure	
Asbestos	Chronic inflammation due to inhalation of asbestos fibers	NLRP3 inflammasome activation, DNA methylation	Pulmonary fibrosis, vascular remodeling, sustained inflammation	[34, 38]
	Proliferation of vascular smooth muscle	Increased expression of Rab5a	Reduced vascular compliance, hypoxia, and vasoconstriction	
Chronic exposure to high altitude	Hypoxia-driven changes in pulmonary artery cells	miRNA gene regulation, DNA methylation, and histone modifications	Increased proliferation, inflammation, fibrosis, and vascular remodeling	[36]
	Changes in miRNA levels (e.g., miR-17, miR-21, miR-190)	Upregulation of miR-17, miR-21, miR-190	Vascular remodeling, decreased vascular compliance	
	Decreased miR-124 expression	Downregulation of miR-124	Fibroblast proliferation and migration contribute to vascular remodeling	
Chronic inflammation	Aging and increased inflammation	Increased DNMT1 and DNMT3a activity	Epigenetic alterations to pulmonary vascular genes leading to fibrosis and vascular remodeling	[12, 37]
	ROS oxidative stress in lungs	Reduced HDAC activity	Sustained inflammation leading to endothelial dysfunction, vascular remodeling	
	Activation of proinflammatory cytokines (IL-6, TNF-α)	Mitochondrial dysfunction, continued ROS production	Pulmonary vascular remodeling, increased pulmonary vascular resistance	
	Mitochondrial dysfunction	Hypermethylation of pulmonary vascular protective genes	Decreased pulmonary compliance, right ventricular hypertrophy	

Roles of key molecular signaling hubs in PH

Bone Morphogenetic Factor Receptor 2 (BMPR2)

BMPR2 is a key player in the BMP signaling pathway, which regulates cell proliferation and apoptosis in vascular endothelial cells and pulmonary artery smooth muscle cells. When cell proliferation becomes excessive, BMPR2 helps inhibit further division and promotes apoptosis to maintain balance [38, 39]. BMPR2 also facilitates the phosphorylation and activation of JNK1, triggering apoptosis. Therefore, its downregulation is likely a contributing factor to apoptosis resistance. Loss of BMPR2, often due to hypermethylation, has been linked to the pathogenesis of PH [40]. Indeed, the downregulation of BMPR2 is implicated in approximately 75% of hereditary PH cases and 26% of idiopathic or immune-related PH cases [41]. Studies have shown that SIN3a, which functions as a scaffold for epigenetic regulators, is shown to repress HDAC activity in PASMCs and may enhance BMPR2 expression in these cells. In PH, loss of SIN3a appears to reduce expression of BMPR2, contributing to pulmonary vascular remodeling.

Transforming Growth Factor Beta (TGF-β)

TGF-β originates from a superfamily of proteins that forms 2 major branches: BMP, discussed above, is part of the growth differentiation factor (GDF) branch, and TGF-β is part of the nodal branch [42]. This study tested the imbalance of BMP GDF branch balance with the TGF-β nodal branch in PH patients and highlighted the shift in favor of the TGF-β nodal branch. This supported the evidence that the reduced/absent expression of BMP due to hypermethylation was associated with PH [39, 40]. Both TGF-β and BMP initiate their effects through binding to AKL type 1 receptors. BMP is most involved with binding to AKL1, whereas TGF-β is most involved with binding to AKL5. When TGF-β binds to AKL5, it signals for fibrosis and proliferation of PASMCs and contributes to remodeling. The study used pharmacological inhibitors of ALK5 in order to restore the TGF-β-BMP balance and found that in doing this, it prevented or reversed pulmonary vascular remodeling in medium-chain triglyceride (MCT)-induced PH in rats. The findings demonstrate that TGF-β activity was dysregulated in PH patients. These findings suggest the potential for targeting the TGFβ nodal and BMP GDF pathways to effectively treat PH. In PH patients, hypermethylation-induced loss of BMPR2 signaling may, therefore, tip the signaling balance toward the pro-fibrotic TGFβ‑ALK5 axis.

NOTCH Receptors

NOTCH2 and NOTCH3 are a group of cell receptors from the NOTCH family (NOTCH1-4), predominantly found on vascular smooth muscle cells (VSMCs), where they play crucial roles in cell proliferation and survival [43]. This study highlights that NOTCH3 promotes the survival of PASMCs through a MER/ERK signaling pathway. Notably, platelet-derived growth factor-B (PDGF-B) increases the expression of NOTCH3, enhancing this survival pathway. In contrast, NOTCH2 appears to have the opposite effect by inhibiting the survival and proliferation pathway, with PDGF-B reducing its expression. The balance between these two receptors may significantly influence the development and progression of PH. While NOTCH3 has been recognized as a marker of cancer, it is now being seen in increased expression on VSMCs, with several rodent models demonstrating a correlation between elevated NOTCH3 levels and the severity of PH [44]. Additionally, the depletion of elastin is associated with epigenetic modifications, specifically DNA methylation, which can upregulate the NOTCH signaling pathways, further contributing to vascular remodeling in PH [45]. These findings suggest that NOTCH3 overexpression may maintain pulmonary artery smooth‑muscle proliferation, whereas NOTCH2 may inhibit it.

Endothelin (ET)

ET-1 (ET-1) is a peptide produced by endothelial cells, VSMCs, and cardiac myocytes, known to function as a potent vasoconstrictor [46]. It is highly expressed in PH and contributes to the progression of the disease through the promotion of inflammation, fibrosis, and vascular remodeling. One study shows that the vasoconstrictor effects of ET-1 are mediated through two types of receptors: ET-A and ET-B. ET-A, as well as cell growth and electrical remodeling, contributes to the proliferation of VSMCs and the thickening of the arterial wall [42]. In contrast, ET-B is primarily responsible for the clearance of ET-1, inhibition of apoptosis, and the release of vasodilators such as NO and prostacyclin. In the study, monocrotaline-induced PH in rabbits demonstrated that the ET-B-mediated NO pathway was not activated in the PH group, while ET-A receptor activation was upregulated, leading to vasoconstriction and vascular remodeling. Furthermore, ET-1 was found to activate the ROCK pathway (Rho-associated coiled-coil containing protein kinase), which contributes to pulmonary vascular remodeling and smooth muscle cell contraction. Inhibition of the ROCK pathway reduced these effects, highlighting its potential as a therapeutic target in PH [47]. These findings suggest that ET‑A receptor activation may promote vasoconstriction and fibrosis, whereas ET‑B clears ET‑1 and is downregulated in PH.

Platelet-Derived Growth Factors

PDGF is another molecule shown to be involved in the promotion of proliferation and migration of PASMCs [48]. This study confirmed this link between PDGF and PASMC cell division and that it may be a factor in pulmonary vascular remodeling. The mechanism involves nuclear factor of activated T-cells (NFATs), regulated by PDGF, which increases the transcription of inflammatory mediators and activates both B- and T-cells. Such activation of lymphocytes and inflammatory mediators may contribute to chronic inflammation and the progression of PH.

Immune-Mediated PH 

There is growing evidence that immune dysregulation may be a contributing factor to the progression of PH. Studies have shown PH is one of the pulmonary complications that affects systemic lupus erythematosus (SLE) patients [49]. PH is reported to occur in approximately 5% to 15% of patients with SLE, although higher rates are reported in echocardiography-screened cohorts [50]. An exact etiopathogenesis for this association is still unknown; it is more common in patients already predisposed to PH from other factors such as genetics and environmental mechanisms. Like IPH, SLE-driven PH is characterized by the proliferation of PASMCs and fibrosis. During SLE, autoantibodies (antinuclear Abs) are deposited in the pulmonary vasculature. These deposits can progress to vasculitis, which contributes to vascular remodeling and the formation of plexiform lesions, which are complex vascular structures commonly associated with PH. Some SLE patients with PH present with a similar vasculopathy to that seen in scleroderma, which is marked by a non-inflammatory pulmonary vascular remodeling and linked to anti-U1 ribonucleoprotein (anti-U1 RNP) positivity. Additionally, overexpression of growth factors and chemokines in the pulmonary arteries further exacerbates the disease process. Although these immune-mediated mechanisms suggest that immunosuppressive therapies could be effective, current evidence remains inconclusive, underscoring the need for further research to determine their efficacy in SLE-related PH. 

Studies have revealed several key molecular pathways contributing to the development of PH in patients with SLE. One study reports that the type I interferon (IFN) response, apoptosis, and protein ubiquitination play critical roles in SLE-related PH [51]. In that regard, abnormal activation of T-cells, a significant source of IFNs, has been linked to inflammation and vascular remodeling, which are key features of the disease. Additionally, the ubiquitin-proteasome system (UPS) has been implicated in the excessive proliferation of PASMCs, with proteasome inhibitors showing potential as a treatment for controlling PH. 

One study supports the link between PH and SLE, highlighting the associations of PH with other connective tissue diseases (CTDs), such as systemic sclerosis (SSc), mixed CTDs (MCTDs), and undifferentiated CTDs (UCTDs) [51]. This study reports that the mortality rate of patients with SSc-associated PH was significantly higher compared to those with UCTD-PH and other CTDs. One notable functional difference was observed in the diffusion capacity of the lungs for carbon monoxide (DLCO), a test that measures gas exchange efficiency. PH is commonly associated with reduced DLCO due to diminished pulmonary blood flow. The lower DLCO results in SSc compared to other CTDs suggest more advanced disease progression and greater impact on pulmonary vasculature in SSc-associated PH. Supporting this, further studies explored the involvement of immune cells and autoantibodies in PH and identified specific autoantibodies as markers for the disease [52]. Notably, anti-centromeric protein autoantibodies found in SSc are associated with proinflammatory cytokine release and vascular remodeling, correlating with higher systolic pulmonary arterial pressure and reduced DLCO. Conversely, the anti-U1 ribonucleoprotein antibody, present in SSc and other CTDs, has protective effects and may be linked to lower mortality in these conditions. Additionally, anti-endothelin receptor type A and anti-angiotensin receptor type 1 antibodies serve as prognostic markers in SSc-PH, contributing to the disease by enhancing vascular endothelial reactivity and promoting pulmonary vasculopathy. Further investigation revealed that IgG anti-fibroblast antibodies in the serum of SSc patients induced the production of proinflammatory cytokines and growth factors, which can facilitate vascular remodeling and the progression of PH. In patients with idiopathic PH, autoantibodies targeting vascular wall components were observed. The production of these antibodies is supported by cytidine deaminase, which plays a role in class switching and somatic hypermutation of antibodies. Moreover, follicular helper T cells (IL-21+PD1+) and CD138+ plasma cells were found to accumulate in remodeled pulmonary blood vessels with antibody deposition, suggesting a significant autoimmune component in IPH. 

Cytokine Networks

Several cytokines, including IL-6, are also involved in the PASMC remodeling and progression of PH. Once IL-6 binds with the IL-6R, which forms a complex with GP130, this complex activates JAK to the phosphorylation of GP130. STAT3 is then recruited and phosphorylated by STAT3, which forms dimers and relocates to the nucleus, promoting the transcription of genes involved with PASMC proliferation, anti-apoptosis pathways, inflammatory genes involved with the inflammasome, and fibrosis [53]. This study found that the administration of IL-6 ligand or the overexpression of a specific IL-6-inducing factor led to the development of PH in mouse models and also found that mice deficient in IL-6 were resistant to hypoxia-induced lung inflammation and PASMC remodeling [49]. The study also reports that using approved treatments that bind to the IL-6R improved severe PH symptoms in patients with mixed connective tissue diseases, but further research is being conducted to assess efficacy and safety. While IL-6 may be a promising therapeutic target in PH, it should also be noted that blocking all IL-6 may dysregulate homeostasis and immunity, meaning that further research may need to be conducted to improve the specificity of treatments. The theory that TGF-B (discussed above) plays an essential role in PASMC remodeling and PH progression, along with IL-6, which, when activated, will up-regulate STAT3 [54]. This study also investigated the effects of miR-125a-5p (a microRNA molecule that negatively regulates tone expression of TGF-B and IL-6 pathways) expression in the development of PH. The study reported that miR-125a-5p was downregulated in PH rat models, and when miR-125a-5p was administered via a nebulizer, it slowed the progression of PH and reduced PASMC remodeling. The downstream effect of this, as seen on histological examination, was that it resulted in inhibited proliferation and promotion of apoptosis in PASMCs. The results mean that miR-125a-5p may have a therapeutic role in the treatment of PH, and there may be other similar molecules that may also be used. 

One study used MCTs to induce PH in rat models. This approach has relevance to the lung injury associated with inhaling essential oils during vaping. This research showed that caspase-8 (and its cleaved form) was significantly upregulated in lung tissue, suggesting a role in apoptosis and potential vascular remodeling [55]. Caspase-8, known as an apoptosis inducer, triggers a downstream caspase cascade, including the activation of caspase-3, which promotes cellular events like membrane blebbing, DNA fragmentation, and ultimately, apoptosis via the extrinsic pathway. In the intrinsic pathway, caspase-8 also cleaves the pro-apoptotic protein Bid into truncated Bid (tBid), which translocates to the mitochondria, induces cytochrome c release, and activates caspase-9, amplifying the apoptotic signal. Upon activation by TNF-α, caspase-8 typically initiates apoptosis. However, when activated by M1 macrophages (M1 MQs), it appears to be involved in VSMC remodeling. Inhibition of M1 MQ-induced caspase-8 activation in the study led to a reduction in SMC proliferation, whereas blocking M2 MQs had no effect, suggesting that caspase-8 may facilitate vascular remodeling and PH when activated by M1 MQs [55]. 

IL-1β is another cytokine reported to be significantly elevated in PH models, which also stimulated PASMC proliferation in vitro. Caspase-8 was also shown to activate caspase-1 via NLRP3, leading to cleavage of pro-IL-1β into active IL-1β. After caspase-8 inhibition, IL-1β levels significantly decreased. Other studies further supported these findings, highlighting the same relationship between caspase-8 and IL-1β, and noted that IL-1β, expressed by neutrophils, also contributes to pulmonary vascular remodeling [56]. The complex interplay between these pathways suggests another potential therapeutic target for modulating PH. 

Chemokines are a more specific subtype of immune cytokines that regulate the actions of cytokines. Some chemokines appear to play roles in the progression of PH. CCL5 is one such chemokine characterized by two adjacent cysteine residues, belonging to the CC chemokine family, and it facilitates the activation and recruitment of immune cells such as T-cells, eosinophils, basophils, monocytes, and dendritic cells. One research group reports that current PH treatments predominantly target vasodilation and fail to address the underlying pathological remodeling driving the disease [57]. This study demonstrates that CCL5 is primarily produced by T-cells and natural killer (NK) cells, which are pivotal in sustaining inflammation in PH. This finding aligns with the literature, which explored the connection between HIV and PH and identified elevated levels of CCL5 as a contributing factor [55]. An interesting aspect is that the human immunodeficiency virus (HIV) targets dendritic cells, macrophages, and CD4+ T-cells, using chemokines to trigger chronic inflammatory responses that can exacerbate PH. Further research has shown that CCL5 binding to its receptor CCR5 on macrophages may activate macrophage recruitment and PASMC proliferation, contributing to PH pathology [25]. This study also highlighted that CCL5 may promote platelet activation, leading to endothelial cell injury and further vascular remodeling [25]. CCL5 deficiency restored BMPR2 signaling and reversed hypoxia-induced remodeling, supporting the concept that targeting the CCL5 pathway could offer a therapeutic strategy for PH. Taken together, these findings suggest that various cytokine network loops like IL‑6/CCL5, IL‑6/STAT3, and CCL5/CCR5 may sustain inflammation, recruit immune cells, and reinforce remodeling in PH.

Summary

**Figure 3:**
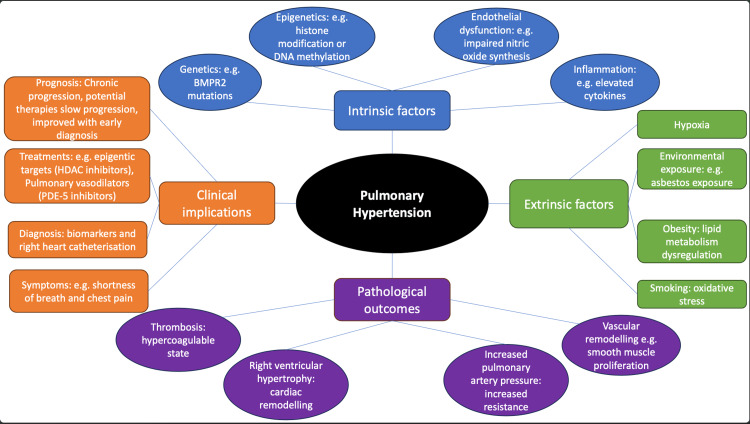
Epigenetic factors in the evolution of pulmonary hypertension (PH) Summary of mechanisms showing interactions involving both intrinsic factors (e.g., genetic mutations and cellular dysfunction) and extrinsic factors (e.g., environmental exposures and lifestyle influences) to drive epigenetic changes in PH.

Figure 3 summarizes the key themes of this review, showing how intrinsic factors such as genetic mutations and cellular dysfunction, and extrinsic factors such as environmental exposures and lifestyle influences, interact to drive epigenetic changes.

## Conclusions

Whereas this review addresses PH broadly, the majority of mechanistic and epigenetic insights discussed are derived from PAH, one of the subtypes of PH identified in this review as Group 1 PH, characterized by intrinsic remodeling of the pulmonary arteries. This review recognizes and acknowledges that the mechanisms discussed may overlap with multiple subtypes of PH. PH is increasingly understood as a multi-hit disorder in which genetic susceptibility and environmental/metabolic stressors converge through epigenetic reprogramming to drive endothelial dysfunction, inflammation, smooth-muscle proliferation, fibrosis, rising PVR, and progressive RV strain. This framework helps explain clinical heterogeneity and highlights actionable upstream drivers, such as oxidative stress, hypoxia, metabolic shifts, and inflammatory signaling, that are not fully addressed by current vasodilator-centric therapies. Emerging strategies targeting epigenetic regulators (e.g., DNMT, HDAC, and BET pathways) and microRNA networks, combined with guideline-based care and exposure/comorbidity management such as smoking cessation, weight optimization, and mitigation of occupational toxins, may guide future efforts to achieve earlier, more personalized intervention. Continued validation of methylation, non-coding RNA, and mitochondrial epigenetic biomarkers and development of safe, selective epigenetic modulators will be essential to translating these mechanistic insights into clinically meaningful advances in the prevention, diagnosis, and treatment of PH. 
